# Genetic association of human Corticotropin-Releasing Hormone Receptor 1 (CRHR1) with Internet gaming addiction in Korean male adolescents

**DOI:** 10.1186/s12888-018-1974-6

**Published:** 2018-12-20

**Authors:** Jooyeon Park, Jin-Young Sung, Dae-Kwang Kim, In Deok Kong, Tonda L. Hughes, Nahyun Kim

**Affiliations:** 1 College of Nursing, Keimyung University, Daegu, Republic of Korea; 2 Department of Medical Genetics, School of Medicine, Keimyung University, Daegu, Republic of Korea; 3 Department of Physiology, Wonju College of Medicine, Yonsei University, Wonju, Republic of Korea; 4 School of Nursing and Department of Psychiatry, Columbia University, New York City, USA

**Keywords:** Internet gaming addiction, Gene polymorphism, *CRHR1*

## Abstract

**Background:**

The number of people with Internet gaming addiction (IGA) is increasing around the world. IGA is known to be associated with personal characteristics, psychosocial factors, and physiological factors. However, few studies have examined the genetic factors related to IGA. This study aimed to investigate the association between IGA and stress-related genetic variants.

**Methods:**

This cross-sectional study was conducted with 230 male high school students in a South Korean city. We selected five stress-related candidate genes: *DAT1, DRD4, NET8, CHRNA4,* and *CRHR1*. The *DAT1* and *DRD4* genes were genotyped by polymerase chain reaction, and the *NET8, CHRNA4,* and *CRHR1* genes were genotyped by pyrosequencing analysis. We performed a Chi-square test to examine the relationship of these five candidate genes to IGA.

**Results:**

Having the AA genotype and the A allele of the *CRHR1* gene (rs28364027) was associated with higher odds of belonging to the IGA participant group (*p* = .016 and *p* = .021, respectively) than to the non-IGA group. By contrast, the *DAT1, DRD4, NET8, and CHRNA4* gene polymorphisms showed no significant difference between the IGA group and control group.

**Conclusions:**

These results indicate that polymorphism of the *CRHR1* gene may play an important role in IGA susceptibility in the Korean adolescent male population. These findings provide a justification and foundation for further investigation of genetic factors related to IGA.

## Background

As the Internet grows, it provides faster and easier ways for people to access information and all types of media. Also expanding is the potential for overuse of the Internet such as online games that may lead to serious addiction. In particular, Internet gaming addiction (IGA) is one of the most serious public health issues among adolescents worldwide. IGA is a subtype of Internet addiction (IA) [1] that has become a socio-pathological phenomenon due to its negative personal and societal consequences. Additionally, IGA shares certain similarities in neurobiological features, personality traits, and behavioral characteristics with substance and behavioral addictions [2, 3].

For these reasons, Internet gaming disorder (IGD), also referred to as IGA, was formally recognized as a potential mental health disorder and was included in section III of the *Diagnostic and Statistical Manual of Mental Disorders* (DSM-V) as a condition warranting further study [4]. In the DSM-V, the classification of IGD is similar to that of pathological gambling and contains nine descriptive criteria: preoccupation; withdrawal; tolerance; loss of control; giving up other activities; continuation; deception; escape; and loss of significant relationships, job, or educational or career opportunities. However, lack of standardized definitions and diagnostic criteria for IGD has hampered progress on research of this condition [1, 2]. Thus, additional research is necessary to establish clear criteria for IGD diagnosis. In this paper, we focus on IGA rather than IGD because it is somewhat narrower in scope and is commonly referred to in the research literature.

IGA is known to be associated with several aspects of individuals’ personal characteristics, psychosocial factors, and neurobiological factors. Personal and psychosocial factors include, for example, age, gender, personality traits, self-esteem, stress, and depression [5–9]. As to neurobiological factors, studies have shown that the dopamine system, which is frequently assessed for addictive disorders, is involved in learning and reinforcement of behaviors. For example, dopamine levels have been associated with pleasure, novelty-seeking behavior, and reward dependency [10]. According to Weinstein and Lejoyeux [11], the level of dopamine release in the ventral striatum during online gaming is comparable to that induced by a substance such as amphetamine. Thus, IGA may be associated with the dopaminergic system in a fashion similar to substance-related addiction. In addition, stress has been reported to be one of the most important factors in addiction [12, 13]. Hypothalamic-pituitary-adrenal (HPA) axis is an intertwining of the central nervous system and endocrine system. Stress stimulates the release of corticotropin-releasing hormone (CRH) from the hypothalamus, and the HPA axis is driven by CRH signaling via *Corticotropin releasing hormone receptor type 1 (CRHR1)* gene. CRH stimulates secretion of adrenocorticotropic hormone (ACTH), and ACTH stimulation produces cortisol. Cortisol triggers negative feedback in the HPA axis [14]. In a recent study, serum cortisol levels were shown to be higher in excessive Internet game users than in non-excessive users [15]. In another study, serum cortisol levels were negatively correlated with severity of pathological gambling [16]. Although these findings are not entirely consistent, addiction is commonly related to HPA axis activity in study results.

Regarding addiction and genetic factors, persistent behavioral changes induced by addiction may alter long-lasting gene expression, significantly contributing to the addiction phenotype [17]. Additionally, pharmacotherapy, a crucial strategy for the treatment of addiction, relies on genetic variation [18, 19]. According to previous studies, IGA seemingly shares some of the features of substance and behavioral addictions not only at the neurocircuitry, psychological, and behavioral levels but also at the genetic level [3, 20].

The majority of studies conducted to evaluate the association between genetic factors and IA or IGA have focused on genes of the dopamine system. A 2007 study found that Taq1A1 allele of the *dopamine D2 receptor (DRD2*) gene was more prevalent in an excessive online gaming group than in a control group [21]. The *dopamine transporter 1 (DAT1)* gene has been shown to be associated with pathological gambling [22]; however, Kim et al. [23] found that the *DAT1* gene was not associated with IA. Given these inconsistent findings, further evaluation is needed to clarify the association between the *DAT1* gene and IGA.

In addition to dopaminergic genes, stress-related hormone genes, including *Catecholamine-O-Methyltransferase (COMT), serotonin transporte*r (5HTTLPR), and *nicotinic acetylcholine receptor subunit alpha 4* (*CHRNA4*) genes have also been examined as potential associated factors with addiction. For example, the low-activity Val158Met variant of the *COMT* gene was more prevalent in an excessive online gaming group than in a control group [21]. Another study reported that the homozygous short allelic variant of the *5HTTLPR* gene was more prevalent in an excessive Internet use group than in a control group [24]. In addition, in Montag’s study [25], an IA group demonstrated significantly more T - (CC) genotype of rs1044396 polymorphism in the *CHRNA4* gene than a control group, and Montag suggested that IA might be partially explained by genetic differences. However, these results were not consistent with those of previous studies [23].

In recent years, the relevance of CRH systems to addictive disorders has received increased attention from researchers. The CRH pathways contribute to the withdrawal/negative affect and preoccupation/anticipation stages of the addiction cycle, and pharmacotherapeutic agents for the CRH systems may be effective in treating some key aspects of addiction [26]. The *CRHR1* gene is involved in the anxiogenic action of CRH [27], and studies have shown that the *CRHR1* gene was associated with addiction vulnerability and modulated the effects of exposure to childhood abuse on addictive disorders such as alcohol use disorder [14, 28, 29]. Although research has been conducted on changes in CRH pathways in relation to IGA [15, 16], few studies have analyzed genetic factors related to CRH systems.

In an effort to address some of the gaps in the current literature we investigated genetic variations associated with IGA. Specifically, we adopted an approach involving gene selection from stress-related genetic variants whose association with IGA has not been previously examined, including *Dopamine Receptor D4* (*DRD4), DAT1, Norepinephrine Transporter 8 (NET8), CHRNA4,* and *CRHR1* genes. These five genes are believed to exert effects on gaming behaviors through regulation of dopamine, norepinephrine, and cortisol levels. We then examined genetic variations of the selected five genes in the IGA and non-IGA groups, hypothesizing that these variations would differ between the two groups.

## Methods

### Participants

**Fig. 1:**
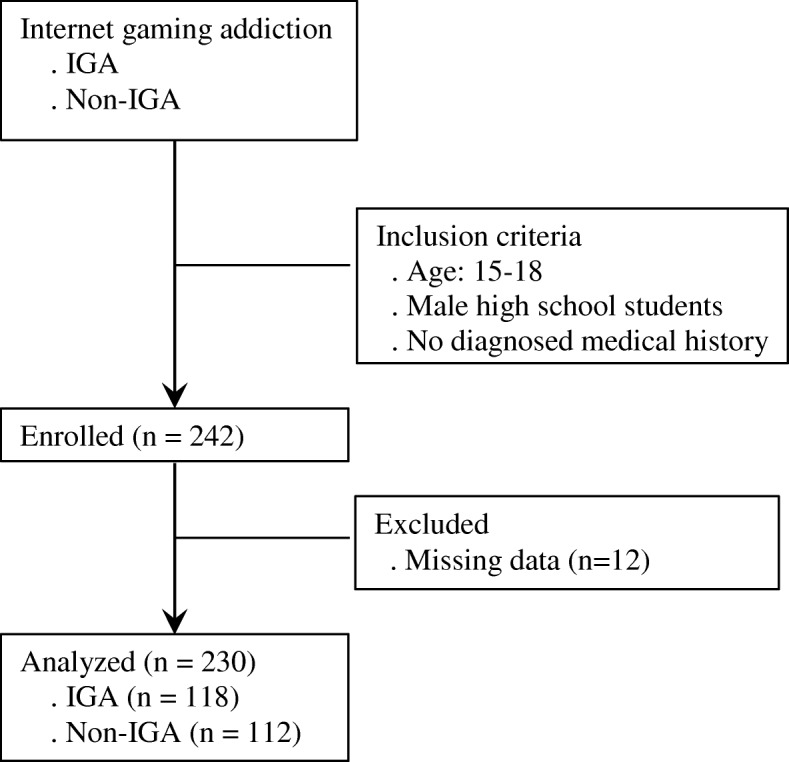
Diagram depicting the selection and flow of participants in the study. IGA, Internet gaming addiction; Non-IGA, non-Internet gaming addiction

This study was a part of larger project that examined the role of the autonomic nervous system in development of IGA among adolescent males [15, 30, 31]. The current study focuses on identifying genomic differences between adolescent males with and without IGA. The 242 participants were 15- to 18-year-old boys recruited from nine high schools in a South Korean city using convenience and snowball sampling methods. We visited each high school to explain the study’s purpose and procedures and invited interested students to participate. We also asked interested students to visit the city’s public sports center—a hub for data collection—on a specific date for collection of study data. To maximize the sample size, we asked the students recruited to invite Internet game-using peers to come with them to the sport center, where we screened them for study eligibility. A minimum sample size of 110 for each of the two groups was estimated for the Chi-square test in order to analyze the genotype frequency between the two groups based on an effect size of 0.3, an alpha level of 0.05, and a power of 0.80 using the G-power program [32]. The study sample was limited to males belonging to only one ethnic group--Korean--because IGA is more common among male than female adolescents [9] and because there may be gender and ethnicity differences in genetic polymorphism [33, 34]. We excluded students with a diagnosed medical condition, including any kind of physical or psychiatric distress, and those taking medications that might affect HPA axis physiology and/or genetic stability (e.g., β-blockers or sedatives). Participants with missing data for any of the study variables were also excluded, and the final study sample consisted of 230 male students (118 with IGA and 112 without). The selection procedure for the study participants is depicted in Fig. 1. Study data were collected in a public sports center. Participants first completed a questionnaire in a private room at the sports center and then provided a blood sample. After completing data collection, we provided participants with health counseling and physical fitness measurements as rewards for participating in our study.

### Measures

#### Internet gaming addiction

Participants were screened using the online game addiction scale for adolescents, which was developed by the Korea Agency for Digital Opportunity and Promotion (KADO) [35]. The scale has established reliability and validity and has been used to screen for IGA among Korean adolescents in national surveys. The scale consists of 20 questions with responses on a 4-point Likert scale ranging from 1 (not at all) to 4 (always). Total scores range from 20 to 80, with higher scores reflecting a greater tendency toward addiction. A score of 49 or above indicates high IGA risk, and a score of 38 or above indicates overuse of Internet games and potential IGA risk [34]. Korean adolescents with high IGA risk and with potential IGA risk share most core characteristics of Internet-related addiction, such as daily life disturbance, withdrawal, tolerance, and preference for the virtual world [34, 36]. Based on KADO scale cut-off scores, our study participants were assigned to either the non-IGA group (scores < 38) or the IGA group (scores > 38). The scale’s Cronbach’s alpha in the current study was 0.93.

#### DNA extraction

Whole blood was collected from participants into tubes containing Ethylenediaminetetraacetic acid (EDTA), and blood samples were preserved in a deep freezer. Genomic Deoxyribonucleic acid (DNA) was extracted directly from the blood samples using standard procedures. After thawing the frozen blood at room temperature, the DNA was extracted from whole blood using a QIAamp Blood Kit (Qiagen, Hilden, Germany) in accordance with manufacturer instructions. The DNA was eluted in 50 μl (μL) of Tris- Ethylenediaminetetraacetic acid (TE) buffer (10 mM Tris-HCl, 1 mM EDTA, PH 8.0) and amplified using a C1000 thermal cycler (Bio-Rad, Hercules, USA).

#### Selection of polymorphism of candidate genes

We selected five genes that were likely to exert an effect on Internet gaming through regulation of dopamine, norepinephrine and cortisol: the *DRD4* variable number of tandem repeats (VNTR) polymorphism*, DAT1* VNTR polymorphism, *NET 8* (rs5569), *CHRNA4* (rs1044396) and *CRHR1* (rs28364027) genes. The selected genes have previously been reported to contain polymorphic variants associated with addiction. The genotype distribution of the five Single nucleotide polymorphisms (SNP) did not deviate from Hardy–Weinberg equilibrium (HWE).

#### Genotyping

The VNTR polymorphisms in the *DRD4* and *DAT1* genes were amplified using polymerase chain reaction (PCR) primers [37, 38]. To determine the repeat numbers of the different alleles of both genes, PCR products were evaluated by means of electrophoresis on ethidium bromide-stained agarose gels.

The *NET8, CHRNA4, and CRHR1* genes were first amplified using PCR primers. Primers were designed with Pyrosequencing Assay Design Software V1.0.6 (Biotage AB, Uppsala, Sweden); a full list of primer sequences and annealing temperatures for each PCR reaction is provided in Table 1. All PCR reactions were checked on a 2.0% agarose gel to ensure successful amplification and specificity before pyrosequencing. Thereafter, genotype was determined by means of pyrosequencing on a PyroMark Q24 (Biotage AB, Uppsala, Sweden). For the pyrosequencing analysis, PCR products were processed in accordance with the manufacturer’s standard protocol (Biotage). A 20 μL PCR template was pipetted into a PSQ 24 Plate Low (Biotage AB, Uppsala, Sweden) containing 2 μL streptavidin-coated Sepharose beads (Streptavidin Sepharose High Performance, GE Healthcare Bio-Science AB, Uppsala, Sweden), 40 μL 2X binding buffer [10 nmol/l Tris (hydroxymethyl)-aminomethan, 2 mol/l NaCl, 1 mmol/l EDTA and 0.1% polyoxyethylenesorbitan monolaureate (Tween 20), pH 7.6], and 18 μL high-performance liquid chromatography-purified water. This mixture was incubated for 10 min at room temperature (shaker speed 1400 rpm). The complexes were purified and separated from the non-biotinylated strands using a PyroMark Vacuum Prep Worktable (Biotage). The beads were then suspended with 20 μL of annealing buffer (20 mM Tris-acetate and 2 mM Mg-acetate at PH 7.6) containing 0.8 μL of sequencing primer. The template-sequencing primer mixture was transferred onto a PSQ 24 Plate (Biotage), heated to 85 °C for 2 min, and cooled to room temperature. Sequencing reactions were performed with a PyroMark Gold Q24 Reagent Kit (Qiagen, Germantown, MD, USA) in accordance with the manufacturer’s instructions.

**Table 1:** PCR primers, PCR conditions and sequencing primers for genotyping five gene using pyrosequencing

	Primer for PCR		Sequencing primer for Pyrosequencing	Annealing temperature
*DRD4* VNTR	Forward	5’-GCGACTACGTGGTCTACTCG-3′	–	60 °C
	Reverse	5’-AGGACCCTCATGGCCTTG-3′		
*DAT1* VNTR	Forward	5’-TGTGGTGTAGGGAACGGCCTGAG-3′	–	65 °C
	Reverse	5’-CTTCCTGGAGGTCACGGCTCAAGG-3’		
*NET8* (rs5569)	Forward	5’-B-GTGAAGAGTTTCCGGTGTCGC-3’	5’-GCATGGAGGCTGTCATC-3’	57 °C
	Reverse	5’-GAGGCATGGAGGCTGTCA-3’		
*CHRNA4* (rs1044396)	Forward	5’-CAGCCCTCTCCGTGCAAAT-3’	5’-CTCTTCGGTGTCCCC-3’	57 °C
	Reverse	5’-B-GGTGCTGCGGGTCTTGAC-3’		
*CRHR1* (rs28364027)	Forward	5’-B-AGGAACCCTGGAGACAGAAGT-3’	5′-GGTTAAGTGAGGGGAA-3’	55 °C
	Reverse	5′-TGGTATGGGGTGGTTAAGTGA-3’		

*PCR* polymerase chain reaction, *B* biotin

### Ethical considerations

This study was approved by the Institutional Review Board of a university in South Korea. Informed and written consent was obtained from all participants as well as their legal guardians prior to data collection.

### Data analysis

Statistical analysis was performed to examine the potential relationships between IGA and SNPs. Data were analyzed using IBM SPSS statistics ver. 20.0 (IBM Co., Armonk, NY, USA). During the analysis, χ^2^-tests were performed to check for significant differences between the IGA and non-IGA groups for HWE. Also, the χ^2^-tests were carried out to identify differences in genetic polymorphism, including genotype and allele frequency, between the IGA and non-IGA groups. An uncorrected *p* value of < .05 was adopted as the threshold for statistical significance.

## Results

Participants’ demographic characteristics, with the exception of daily sleep time, did not significantly differ between the IGA and non-IGA groups. The mean age of the participants was 16.63 years, with ages ranging from 15 to 18 years, and their mean body mass index (BMI) was 21.91 kg per square meter (kg/m^2^), with values ranging from 15.4 to 36.7 kg/m^2^. About one-quarter of the participants reported that they currently smoked cigarettes and drank alcoholic beverages; the percentage was similar in the IGA and non-IGA groups. Perceived academic performance was also similar in the two groups. However, daily sleep time was significantly shorter in the IGA group, with about one-third of the participants indicating that they slept less than 6 h per day. Regarding Internet gaming-related characteristics, participants’ daily Internet gaming time averaged 171.96 min (227.29 min in the IGA group and 113.66 min in the non-IGA group), their average duration of Internet gaming was 7.21 years (7.64 years in the IGA group and 6.82 years in the non-IGA group), and their average IGA score was 36.50 (46.05 in the IGA group and 26.43 in the non-IGA group). All these Internet gaming-related characteristics significantly differed between the IGA and non-IGA groups. Additional details of the participants’ demographic and Internet gaming-related characteristics are provided in our previous study [31].

As shown in Table 2, the 48 base pair (bp) VNTR polymorphism in the *DRD4* gene was classified as long alleles (repeats ≥5) and short alleles (repeats < 5) based on a previous study [23]. The homozygous short allelic variant of the *DRD4* gene VNTR polymorphism was the most common in the IGA and non-IGA groups. The *DAT1* gene VNTR polymorphism showed 7- to 11-repeat alleles of 40 bp, and the 10-repeat allele was approximately 94% in both groups. There was no significant difference in the *DRD4* and *DAT1* VNTR polymorphisms between the IGA and non-IGA groups.

**Table 2:** *DRD4* and *DAT1* VNTR genotypes and allele frequencies between IGA and non-IGA groups

Gene	Genotype	IGA (*n* = 116)	Non-IGA (*n* = 114)	χ^2^1	*p*1	Allele type	IGA (*n* = 116)	Non-IGA (*n* = 114)	χ^2^2	*p*2
		*n* (%)	*n* (%)				*n* (%)	*n* (%)		
*DRD4*	l/l	0 (0.0)	0 (0.0)	0.32	.571	l	2 (0.9)	1 (0.4)	0.31	.573
	l/s	2 (1.7)	1 (0.9)			s	230 (99.1)	227 (99.6)		
	s/s	114 (98.3)	113 (99.1)							
*DAT1*	7/10	4 (3.4)	5 (4.4)	2.11	.806	7-repeats	4 (7.1)	5 (2.2)	1.05	.787
	9/10	7 (6.0)	4 (3.5)			9-repeats	7 (3.0)	4 (1.8)		
	10/10	102 (87.9)	102 (89.4)			10-repeats	218 (94.0)	215 (94.2)		
	10/11	3 (2.7)	2 (1.8)			11-repeats	3 (1.3)	4 (1.8)		
	11/11	0 (0.0)	1 (0.9)							

*VNTR* variable number of tandem repeats, *IGA* Internet game addiction, *non-IGA* non-Internet game addiction; χ^2^1, χ^2^ in comparison of genotype frequency between IGA and non-IGA; *p*1, *p*-value in comparison of genotype frequency between IGA and non-IGA; χ^2^2, χ^2^ in comparison of allele frequency between IGA and non-IGA; *p*2, *p*-value in comparison of allele frequency between IGA and non-IGA; l, 48-base pair repeats ≥5; s, 48-base pair repeats <

As Table 3 shows, the genotype and allele frequencies of the *CRHR1* (rs28364027) gene differed significantly between the IGA and non-IGA groups (χ^2=^5.76, *p* = .016 and χ^2=^5.36, *p* = .021, respectively). The presence of the AA genotype increased the risk of IGA (*odds ratio*: 2.68, 95% *CI*: 1.172–6.149), whereas the presence of G-allele carriers did not. No significant differences in the *NET8* (rs5569) or *CHRNA4* (rs1044396) genes were observed between the IGA and non-IGA groups.

**Table 3:** *NET8* (rs5569), *CHRNA4* (rs1044396) and *CRHR1* (rs28364027) genotypes and allele frequencies between IGA and non-IGA groups

Gene (SNP)	MAF	Genotype	IGA (*n* = 116)	Non-IGA (*n* = 114)	χ^2^1	*p*1	Allele type	IGA (*n* = 116)	Non-IGA (*n* = 114)	χ^2^2	*p*2	*OR* (95% *CI*)
			*n* (%)	*n* (%)				*n* (%)	*n* (%)			
*NET8* (rs5569)	0.25	AA	65 (58.6)	70 (50.2)	0.89	.640	A	177 (76.0)	167 (73.2)	0.50	.450	0.78 (0.47–1.32)
		AG	41 (35.3)	47 (41.3)			G	56 (24.0)	61 (26.8)			
		GG	7 (6.1)	7 (6.1)								
*CHRNA4* (rs1044396)	0.21	CC	68 (58.6)	71 (62.3)	0.59	.730	C	180 (77.6)	180 (78.9)	0.10	.730	1.17 (0.63–1.95)
		CT	44 (37.9)	38 (33.3)			T	52 (22.4)	48 (21.1)			
		TT	4 (3.5)	5 (4.4)								
*CRHR1* (rs28364027)	0.07	AA	107 (92.2)	93 (81.6)	5.76	.016	A	223 (97.8)	207 (94.7)	5.36	.021	2.63 (1.17–6.15)
		AG	9 (7.8)	21 (18.4)			G	9 (2.2)	21 (5.3)			
		GG	0 (0.0)	0 (0.0)								

*IGA* Internet gaming addiction, *non-IGA* non-Internet gaming addiction, *SNP* single nucleotide polymorphism, *MAF* minor allele frequency; χ^2^1, χ^2^ in comparison of genotype frequency between IGA and non-IGA; *p*1, *p*-value in comparison of genotype frequency between IGA and non-IGA; χ^2^2, χ^2^ in comparison of allele frequency between IGA and non-IGA; *p*2, *p*-value in comparison of allele frequency between IGA and non-IGA; *OR*, odds ratio; *CI*, confidence interval

## Discussion

In our study, we investigated the influence of gene polymorphism on IGA risk for five stress-related candidate genes: the *DRD4* VNTR, *DAT1* VNTR, *NET8* (rs5569), *CHRNA4* (rs1044396), and *CRHR1* (rs28364027). We selected a gender- and age-controlled sample to minimize the impact of confounding variables on our genetic findings; in addition, our study was conducted with ethnically homogeneous participants. Notably, we found significant differences in the genotypes and allele frequency of rs28364027 between the IGA and non-IGA groups, but not in those of the other candidate genes.

Specifically, we found no association between 48 bp VNTR polymorphism in the *DRD4* gene and IGA. Our findings are seemingly quite different from study results for Western populations. In Western studies, the 48 bp VNTR polymorphism in the *DRD4* gene has been associated with a number of addictive disorders, including substance abuse [39–41], alcohol dependence [42, 43], and smoking [44]. In particular, the 7-repeat allele of the *DRD4* 48 bp VNTR polymorphism has been reported as posing a risk for addictive disorders in Western populations [39, 40, 42, 44]. However, in Asians, including the Korean participants in our study, the *DRD4* gene has not been associated with addictive disorders [45–47]. The rarity of the 7-repeat allele in Asian populations, including the Japanese, Chinese, and Taiwanese, may have contributed to the inconsistency between our findings and Western study results [34, 48]. On the whole, the genotype distribution of the *DRD4* gene has shown considerable variation by ethnicity. Further research is needed to clarify whether the genotype and allele frequency of the *DRD4* gene polymorphism influences IGA in particular races and ethnicities.

In addition, we found no differences in the genotypes and allele frequencies of the *DAT1*, *NET8,* and *CHRNA4* gene polymorphisms between the IGA and non-IGA groups. The VNTR polymorphism in the *DAT1* gene has been shown to alter *DAT1* gene expression [49], and prior studies have demonstrated that the presence of the 9-repeat allele increased susceptibility to addiction more than alleles having more than 9-repeats [50, 51]. In Western populations, the *DAT1* gene was associated with alcohol dependency [51], substance abuse [52], and pathological gambling [22]. In contrast, we found no association between the 40 bp VNTR polymorphism in the *DAT1* gene and IGA. On the other hand, our results are consistent with the findings of previous studies of Korean populations with addictive disorders--like, alcohol and nicotine use disorders [53, 54].

The *NET8* gene polymorphism was shown to be correlated with reward dependency in Cloninger’s temperament dimensions [55]. Based on this and findings from other previous studies, we hypothesized that the *NET8* gene polymorphism was associated with IGA, but we found no differences in the genotype and allele frequency of the *NET8* gene polymorphism between the IGA and non-IGA groups. Nonetheless, our non-significant findings in this regard provide information about stress-related gene traits that has not previously been reported in IGA studies.

Regarding the *CHRNA4* gene, we found no significant differences in polymorphism between the IGA and non-IGA groups. The *CHRNA4* gene has been reported to impact dopaminergic neurotransmission in animal models [56]; additionally, this gene has been found to be a factor in addictive disorder in several animal and human studies. For example, the CC genotype of rs1044396 in the *CHRNA4* gene was found at a much higher frequency in individuals exhibiting addictive behavior and showed relatively consistent results across ethnicities [25, 57–59]. Similarly, our study indicated that the CC genotype of rs1044396 was present at a higher frequency in the IGA group than in the non-IGA group, but there was not a significant difference. The IGA group’s having a more frequent CC genotype of rs1044396 than the non-IGA group but not significantly so can be explained in several ways. First, prolonged and excessive Internet gaming might activate the HPA axis [60, 61], resulting in CC genotype of rs1044396; however, this pathway did not seem to cause a significant change in our Korean population. Second, our participants’ demographic characteristics might have contributed to the statistical non-significance. Our participants were restricted to male adolescents in high school, whereas Montag et al. [25] employed male and female university students with and without IA in identifying the candidate gene of IA—the *CHRNA4* gene. In Montag’s study, the CC genotype of rs1044396 in the *CHRNA4* gene occurred significantly more frequently in the IA group—specifically, this effect was driven by female gender [25]. Third, our study had a relatively small sample, which may have increased the possibility of false-negative results. Because ours is the first study to reveal an association between variants in the *CHRNA4* gene and IGA, we cannot be certain that statistically significant differences in polymorphism would have been found in a larger sample. Therefore, replication studies with larger samples are needed to determine whether *CHRNA4* gene polymorphism contributes to IGA.

Interestingly, we found that the AA genotype of rs28364027 in the *CRHR1* gene was associated with IGA, as it was much more frequent in the IGA group than in the non-IGA group. The *CRHR1* gene polymorphism has been primarily associated with stress-related phenotypes, including altered cortisol response [62, 63] and alcohol dependency [64–66]. Kim and colleagues [67] reported that an allele of rs28364027 was associated with alcohol dependency in the Korean population.

Furthermore, we observed that the AA genotype of rs28364027 showed a 2.69 times greater increase in the odds for IGA (95% CI [1.17–6.15]) compared to the G-carrier genotype. According to the literature, the *CRHR1* gene is well known to be related to alcohol dependency, but in our study, alcohol consumption and even smoking rate did not differ between the IGA and non-IGA groups. Among the other covariates, daily sleep time was significantly shorter with IGA than without IGA. However, previous research has provided little evidence that the *CRHR1* gene is related to sleep behavior; rather, circadian clock and serotonin transporter genes have mainly been related to sleep phenotypes [68–70]. Thus, our findings suggest that the *CRHR1* gene is a promising candidate for IGA risk research.

Regarding the genetic differences between individuals with and without a certain addiction, researchers have emphasized the framework of gene-environment (GxE) interaction to explain those variations. For example, an interaction between genetic polymorphism and stressful life events was tentatively identified as a predictor or moderator and triggered alcohol use disorders [14, 28, 29], nicotine dependence [71], and cannabis use [72]. Although a variety of stressors are known to be important risk factors for addiction [12, 13], some researchers have argued that addiction symptoms or addictive behaviors such as cravings [73, 74], immersion [75, 76], withdrawal [77], pathologic gambling [16], and game playing [60, 61, 78] could also produce biological stress responses by activating the HPA axis or sympathetic nervous system [15, 16, 73, 79, 80]. Specifically, studies of Internet/online gaming have reported that the activity of the HPA axis and sympathetic nervous system were increased during and after gaming [15, 60, 61, 78, 79] and even in the resting state in prolonged game users [30, 31].

Based on the results of this and previous studies, we offer two possible explanations for the genetic association of the *CRHR1* gene with IGA in Korean male adolescents. First, the IGA group in the current study had been involved in Internet gaming for 7.21 years on average. Thus, it is possible that Internet gaming itself stimulated the addiction tendency through long-term exposure to gaming and to stress responses induced by gaming. Second, given our findings and those of previous studies, it seems reasonable that an interaction exists between the AA genotype of rs28364027 as a risk factor for IGA and excessive gaming immersion, which constitutes a stress event that reinforces the addictive behavior.

Gene polymorphism is an important concept that explains not only differences in appearance, but also susceptibility to diseases, expression patterns of diseases, and responses to medications [81]. However, environmental factors also play an important role in individual traits, although genetic predispositions affect much of a given trait [82]. In particular, investigation of the relationship between the *CRHR1* gene polymorphism and GxE interaction in IGA is worthy of future study. Such research could inform the development more effective intervention programs by addressing environmental factors based on a greater understanding of genetic predisposition.

We conducted this investigation because no previous studies have examined the potential associations between stress-related genes and IGA, although some studies have reported relationships between these genes and other addictive disorders. Our results should be interpreted in light of the strengths and limitations of our study. The strengths include well-ascertained stress-related phenotypes and a relatively homogeneous sample in terms of age, gender, and Korean-only ethnicity. Moreover, our findings provide a justification and foundation for further investigation of genetic factors related to IGA.

Despite the strengths of our study, some limitations should be acknowledged. First, even though our sample was homogeneous on the whole, our sample size was relatively small, which may have limited the generalizability of our findings. Second, the study design was cross-sectional, and we did not directly examine the function of rs28364027 in the *CRHR1* gene or whether *CRHR1* gene polymorphism and stress events have a GxE interaction in IGA. Therefore, we could not identify a causal association between IGA and the *CRHR1* gene polymorphism. Finally, we did not correct for multiple comparisons, and therefore the associations between variants in the *CRHR1* gene and IGA reported here may be limited to nominal significant associations. Further studies are needed to investigate the potential interaction between the *CRHR1* gene polymorphism and IGA in a larger sample and to identify the functionality of rs28364027. In addition, studies of varying racial and ethnic populations are warranted to better understand potential race/ethnicity-related genetic associations with IGA.

## Conclusions

In summary, ours is the first study to reveal a significant association between variants in the *CRHR1* gene and IGA. The AA genotype of rs28364027 in the *CRHR1* gene was much more frequent in the adolescents with IGA than in those without IGA. Therefore, polymorphism in the *CRHR1* gene may play an important role in IGA susceptibility in the Korean adolescent male population. These findings provide a basis for further investigation of genetic factors related to IGA. In addition, early assessment and intervention for adolescents engaging in excessive Internet gaming are needed to prevent adverse genetic consequences, including epigenetic changes resulting in cardio-metabolic health outcomes (e.g., cardiovascular disease, Type 2 diabetes, and hypertension) and psychiatric distress in adulthood [83, 84].

## Abbreviations

5HTTLPR: Serotonin transporter gene
ACTH: Adrenocorticotropic hormone
BMI: Body mass index
bp: base pair
CHRNA4: Nicotinic acetylcholine receptor subunit alpha 4
CI: Confidence interval
COMT: Catecholamine-o-methyltransferase
CRH: Corticotropin releasing hormone
CRHR1: Corticotropin releasing hormone receptor type 1
DAT1: Dopamine transporter 1
DNA: Deoxyribonucleic acid
DRD2: Dopamine D2 receptor
DRD4: Dopamine receptor D4
DSM-V: The fifth edition of the diagnostic and statistical manual of mental disorders
EDTA: Ethylenediaminetetraacetic acid
GxE interaction: Gene environment interaction
HPA: Hypothalamic pituitary adrenal
HWE: Hardy Weinberg equilibrium
IA: Internet addiction
IGA: Internet gaming addiction
IGD: Internet gaming disorder
KADO: Korean agency for digital opportunity and promotion
NET8: Norepiniphrine8
PCR: Polymerase chain reaction
SNP: Single nucleotide polymorphisms
TE: Tris ethylenediaminetetraacetic acid
VNTR: Variable number of tandem repeats
